# Perioperative psycho-behavioral stress promotes cancer metastasis beyond the impact of surgery: Neuroendocrine and tumor molecular mediating mechanisms

**DOI:** 10.1016/j.bbih.2026.101228

**Published:** 2026-03-26

**Authors:** Bar Bruno Shvalbo, Sharon Scarlat, Nahida Sakis, Estherina Trachtenberg, Elad Sandbank, Meshi Weil, Anabel Eckerling, Steven W. Cole, Shamgar Ben-Eliyahu

**Affiliations:** a School of Psychological Sciences, Tel Aviv University, Tel Aviv, Israel; b Sagol School of Neuroscience, Tel Aviv University, Tel Aviv, Israel; c MRC Cognition and Brain Sciences Unit, University of Cambridge, Cambridge, UK; d Departments of Psychiatry & Biobehavioral Sciences and Medicine, University of California, Los Angeles, USA

**Keywords:** Metastasis prevention, Perioperative period, Psycho-behavioral stress, Propranolol, Beta-blockers, Etodolac, Mifepristone, Stress models, Stress-inflammatory responses

## Abstract

Clinically, the unique impact of perioperative distress on long-term cancer outcomes remains unclear and is thus overlooked medically. Operated cancer patients experience intertwined psychological and physiological stress, which are hard to disentangle. Therefore, herein we employed rodents to assess the specific impact of psycho-behavioral stress on cancer metastasis in the context of surgery and without it, and studied underlying neuroendocrine mechanisms and intratumoral transcriptional mediators. A tilt-light stress paradigm was employed along with surgery/tumor cell inoculation, using two mouse colorectal cancer models of liver metastases (MC38 C57BL/6, and CT26 BALB/c), and a rat mammary pulmonary metastases model (MADB106 F344). In the latter, we also assessed the efficacy of glucocorticoid, β-adrenergic, or COX-2 inhibitors/antagonists (mifepristone, propranolol, or etodolac) in preventing the effects of stress. A 48-h perioperative stress exposure, but not 24-h pre- or 24-h post-operative exposure, consistently increased the number of experimental metastases in all models, irrespective of the effects of surgery. Both mifepristone and propranolol, but not etodolac, prevented these effects. To better mimic the clinical setting, the 4T1 mammary cancer model of spontaneous metastases was used, along with six perioperative days of alternating stress. Perioperative stress increased metastasis, and a propranolol + etodolac regimen, as employed in recent clinical studies, abrogated pre-operative deleterious effects of stress on tumor epithelial-to-mesenchymal transition and other metastasis-related transcriptional activity. Overall, psycho-behavioral stress can uniquely contribute to metastatic progression through stress responses and malignant pro-metastatic transcriptional pathways. Thus, in cancer patients, managing perioperative distress may improve long-term cancer outcomes.

## Highlights

• Forty-eight hr psycho-behavioral stress increased metastases in three rodent models.
• Stress increased metastasis independently of the impact of surgery.
• Mifepristone and propranolol each completely abolished the increase in metastases.
• Stress induced prometastatic tumoral transcriptional activity and EMT.
• A propranolol + etodolac regimen blocked stress-induced transcriptional perturbations.

## 1 Introduction

The short perioperative period, spanning days to weeks before and after surgery, has been recognized as pivotal in determining long-term cancer outcomes, disproportionately to its short duration (Horowitz et al., 2015; Tohme et al., 2017; Matzner et al., 2019). This period is characterized by elevated stress and inflammatory responses, which are tightly related to a multitude of factors, including perioperative psychological distress (Rowe et al., 2025; Ohkura et al., 2020), exposure to anesthetic and analgesic agents (Choi and Hwang, 2024), tissue damage (Santander Ballestín et al., 2022), nociception and pain (Zajączkowska et al., 2018; Jacobs et al., 2024), hypothermia (Sandbank et al., 2024), and blood loss and transfusion (Cata et al., 2013). Surgery-related immunosuppression is an acknowledged robust phenomenon, with an intricate biological underpinning, including activation of the (i) sympathetic nervous system (SNS), (ii) hypothalamic-pituitary-adrenal (HPA) axis, and (iii) inflammatory and anti-inflammatory responses (Neeman and Ben-Eliyahu, 2013; Muthuswamy et al., 2017; Eckerling et al., 2021). These neuroendocrine and paracrine responses also act directly on the malignant tissue and its microenvironment, and have been shown to worsen long-term cancer outcomes (Zahalka and Frenette, 2020; Chang et al., 2022; Khadka et al., 2023). These processes can synergistically promote metastatic progression, while their blockade may shift a sensitive perioperative balance towards an anti-metastatic dominance, which may be supported by tumor removal and other perioperative interventions (Ben-Eliyahu, 2020). Thus, the short perioperative period presents a critical timeframe that can hypothetically be leveraged to improve long-term clinical outcomes in cancer patients through pharmacological and psycho-behavioral approaches (Ben-Eliyahu, 2020; Matzner et al., 2020; Chang et al., 2022; Hanalis-Miller et al., 2024). Indeed, in translational studies, we and others have shown that the separate or combined blockade of catecholamines and prostaglandins, using propranolol and/or etodolac, can reduce surgery-induced metastasis and improve postoperative long-term cancer survival rates (Sorski et al., 2016; Koh et al., 2021; Haldar et al., 2023; Lin et al., 2023).

Consequently, in the last decade, we have treated breast (BC) and colorectal cancer (CRC) patients perioperatively with propranolol + etodolac in two clinical trials, starting 5 days before surgery. The treatment has positively affected multiple biomarkers of metastatic progression. In excised tumors from both trials, the treatment reduced mesenchymal polarization and downregulated the activity of pro-metastatic transcription factors (TFs), including GATA-1 & 2, STAT3, and GRE (Shaashua et al., 2017). In BC patients, treatment also decreased the expression of the tumor proliferation marker Ki-67 (Haldar et al., 2018), and in CRC patients, the treatment improved 5-year disease-free survival (DFS), although the study was not statistically powered for this outcome (Ricon-Becker et al., 2023a).

However, in the clinical context of surgery, it remains unclear whether psychologically induced stress-inflammatory responses have unique deleterious impacts on long-term cancer outcomes, and whether their blockade would be beneficial beyond addressing the physiological effects of surgery (Haldar et al., 2020; Hiller et al., 2020). Oncologic patients experience intertwined psychological and physiological stress, and dissociating the two while pinpointing the long-term repercussions of each is challenging clinically (Antoni and Dhabhar, 2019; Ohkura et al., 2020; Eckerling et al., 2021). Our clinical findings in BC patients do suggest short-term biological impacts of perioperative psychological stress, but not long-term effects. Specifically, in the placebo group, plasma IL-6 and CRP levels increased even before surgery, which can be ascribed to pre-surgical psychological stress, and the drug treatment completely abrogated this heightened inflammatory response (Shaashua et al., 2017). However, whether such hypothesized psychological effects also worsen long-term cancer outcomes in the context of surgery remains largely undetermined (Huang et al., 2025).

Elucidating and potentially recognizing the unique and long-term clinical significance of psychological stress in the context of cancer surgery is important for several reasons. First, psychological stress responses include elevated glucocorticoid levels (Vignjević Petrinović et al., 2023), which have been shown by translational studies to promote metastasis (Matzner et al., 2019; He et al., 2024). However, it is challenging to pharmacologically address HPA responses in the perioperative context (Feng et al., 2024), while psychological interventions are feasible and efficient in limiting glucocorticoid-related responses (Mészáros Crow et al., 2023), including in BC patients (Hanalis-Miller et al., 2024). Second, most patients are currently not eligible for the propranolol + etodolac treatment, due to medical contraindications to these drugs, while psycho-behavioral stress-management interventions may partially constitute alternative approaches (Hanalis-Miller et al., 2024). Last, an integrated psychological-pharmacological approach may be implemented for longer periods before and after surgery without additional risks of drug-related adverse events, consequently targeting a wider range of deleterious effects for a longer duration. Nevertheless, the higher cost of prolonged psycho-behavioral interventions, and the clinical skepticism regarding the long-term oncological significance of psychotherapy (Levett and Grimmett, 2019), constitute robust obstacles for implementing such an integrated psychological-pharmacological approach in the context of surgery. Recent reviews by Antoni et al. and Eckerling et al. suggest that, whereas stress-management interventions could be promising clinically, even at the level of cancer recurrence, further inquiries into the underlying mechanisms are needed, and the timing when maximal beneficial effects of such interventions can be achieved should be further explored (Eckerling et al., 2021; Antoni et al., 2023).

Addressing these issues and the unique impact of psychological stress in the perioperative oncological context is more feasible and methodologically robust in translational studies. Accordingly, the objectives of the current translational study were: (i) to elucidate the long-term impact of psychological stress on cancer progression, beyond the impact of surgery but in its context, (ii) to examine whether there is a short critical period, just before or just after surgery, where psychological stress plays a more significant role in facilitating metastatic development, (iii) to pinpoint neuroendocrine mediating pathways of the impact of stress on cancer metastasis, and (iv) to test the effects of pre-operative psychological stress on biomarkers of metastasis in excised tumors, without involving the robust surgical stress that characterizes the clinical context, and that is a major obstacle for such studies in cancer patients.

## 2 Materials and methods

### 2.1 Animals

Male and female C57BL/6J mice and BALB/c mice, and Fischer 344 (F344) rats were housed 3-4 per cage in same-sex groups. We conducted all experiments when animals reached 3-5 months of age. Food and water were available *ad libitum* under a 12:12 light-dark cycle at 22-24 °C. Throughout the experimental duration, animals were monitored for weight, visible injuries, and general well-being. Weight, drug treatment, and stress exposure were counterbalanced across experimental groups. Rats were handled at least 3 times before each experiment to reduce procedural stress. Housing conditions and experimental procedures were monitored and approved by the Institutional Animal Care and Use Committee of Tel-Aviv University (approval numbers: TAU-LS-IL-2601-111-5, TAU-LS-IL-2601-102-5, TAU-LS-IL-2603-124-5).

### 2.2 Experiments 1-3

#### 2.2.1 Cancer models and surgical procedures

MC38: a murine colon adenocarcinoma syngeneic to C57BL/6J mice (Corbett et al., 1975) (kindly gifted by Dr. Eran Nizri, Tel-Aviv Sourasky Medical Center). Cells were grown in monolayer cultures at 37 °C, 100% humidity, 5% CO2, in DMEM (Dulbecco's Modified Eagle Medium) supplemented with 10% fetal bovine serum (FBS), 1% penicillin-streptomycin antibiotic solution. Cells were removed from the culture flask with a trypsin solution (0.25% in PBS), washed once in PBS with 0.1 mg/ml BSA (335g for 5 min), and adjusted to a final concentration of 1 × 10^5^/ml. For tumor cell inoculation, mice were maintained under 2.5% volatile isoflurane anesthesia. The operated surface was shaved and sterilized using 70% ethanol, and a 0.5 cm long abdominal incision was made above the approximate location of the spleen. Cells were injected at a final volume of 100 μL per animal into the spleen using a 31G needle, which was removed from the spleen 2 min following injection. A 4/0 polypropylene monofilament non-absorbable suture was placed across the hilum of the spleen to prevent bleeding, and a splenectomy was then performed. Following the excision of the injected spleen, the incision was glued, and a local anesthetic was applied (lidocaine 2.5% and prilocaine 2.5% cream). Animals were allowed to recover in their home cages, and paracetamol in drinking water was provided for 24 h (3 mg/ml). Animals were monitored for general well-being and signs of distress. The experiment was terminated 21 days later. Animals were euthanized with volatile isoflurane overdose. Livers were harvested post-mortem, and hepatic metastases (spherical formations over 1 mm in diameter) were counted by an experimenter blind to the experimental conditions.

CT26: a colon carcinoma cell line, syngeneic to the BALB/c strain (Corbett et al., 1975) (kindly gifted by Prof. Eliezer Flesher, Faculty of Medicine, Tel Aviv University). Cells were grown in monolayer cultures in RPMI 1640 supplemented with 10% heat-inactivated fetal calf serum, 50 μg/ml gentamicin, 2 mM L-glutamine, 1 mM sodium pyruvate, and 0.1 mM non-essential amino acids (Biological Industries, Israel), and maintained in the same conditions as the MC38 cell line. Cells were removed from the culture flask with a 0.25% trypsin solution in PBS, washed once in PBS containing 0.1 mg/ml BSA (335g for 10 min), and adjusted to a final concentration of 1 × 10^5^/ml in PBS-BSA. The rest of the experimental protocol was as described above (MC38).

MADB106: a mammary adenocarcinoma cell line syngeneic to F344 rats (Barlozzari et al., 1985). Cells were cultured in a monolayer and prepared for injection as described for the CT26 cell line. Each rat was inoculated with 50,000 cells through the tail vein under a brief 2.5% isoflurane anesthesia, using a 21G needle. Following tumor inoculation, animals were monitored regularly, and the experiments were terminated 18-21 days after inoculation, once signs of illness such as rapid weight loss or unkempt fur were present. At the experiment's endpoint, rats were euthanized by isoflurane overdose. Then, lungs were harvested, placed in Bouin's solution for 24 h (Goldfarb et al., 2009), and washed in ethanol. Thereafter, pulmonary metastases were enumerated by an experimenter blind to the animals' experimental condition.

#### 2.2.2 Stress paradigm

Tilt-light stress: The paradigm was described in detail by Matzner et al. (2019). Briefly, rat or mouse cages were positioned at a 45° angle in a constantly lit room at 18 °C for 24 h, creating a limited floor area and a disturbance of the light-dark cycle. Food and water were available *ad libitum*, and a clean cage with unused sawdust was used for the paradigm. According to our previously published data, this paradigm induces at least a 1.5-fold increase in plasma corticosterone, as measured shortly after paradigm initiation and 16 h later (Matzner et al., 2019).

### 2.3 Experiments 4-6

This series of experiments was conducted in 3- to 5-month-old F344 rats.

#### 2.3.1 Cancer model and stress paradigm

The MADB106 model was used in these experiments. Cells were prepared and injected as described above, 24 h after the beginning of the 48-h tilt-light paradigm (described above).

#### 2.3.2 Pharmacological interventions

Four subcutaneous administrations of either RU-486, propranolol, or etodolac were conducted twice daily (morning and evening), starting in the morning, 30 min before the beginning of the 48-h stress paradigm. The control group in each experiment received an equal amount of vehicle injections. The first dose of each of the substances was based on effective blockade evident in our previous studies (Benish et al., 2008; Rosenne et al., 2014), and the following three doses were calculated based on the known or estimated half-life of each compound in its vehicle, to maintain effective blockade throughout the 48-h stress paradigm.

RU-486 (mifepristone): RU-486 (Sigma, Israel) is an antagonist for glucocorticoid and progesterone receptors. The first dose was 25 mg/kg mifepristone (RU-486) dissolved in 200 μL corn oil. The next three injections were 8 mg/kg in the same volume of corn oil.

Propranolol: propranolol (Sigma, Israel), a non-selective β-adrenergic blocker, was administered at a constant dosage of 1.5 mg/kg in a slow-release emulsion. The slow-release vehicle consisted of 4 parts PBS, 3 parts mineral oil (Sigma, Israel), and 1 part mannide-monooleate (an emulsifying agent, Sigma, Israel).

Etodolac: a semi-selective COX-2 inhibitor (10:1 COX-2:COX-1 (Glaser, 1995)) (kindly donated by Taro, Israel) was administered at an initial dose of 12.5 mg/kg dissolved in 200 μL corn oil, followed by three additional injections of 4 mg/kg in 200 μL corn oil.

### 2.4 Experiments 7

#### 2.4.1 Cancer model

4T1 mammary carcinoma: The 4T1.LUC2 cell line is a naturally metastasizing mammary carcinoma, syngeneic to BALB/c mice. Cells were cultured and prepared as described above for the CT26 cell line. Cells were used to induce primary tumors by orthotopic injection of 1 × 10^5^ cells into the fourth mammary fat pad in a volume of 20 μL per animal using a 30G needle under brief 2.5% volatile isoflurane anesthesia. Mice were monitored, and tumors were measured using a digital caliper. Two weeks after tumor inoculation, during the prolonged stress paradigm (described in Section 2.4.2), tumors were resected from all mice, as in the clinical context, to assess the effect of stress independently of tumor-secreted factors, while minimizing pain from primary tumor growth. For resection, mice were anesthetized using 2.5% volatile isoflurane. The tumor–skin complex was carefully removed with sterilized surgical equipment without injuring the muscle tissue. The operated area was sutured immediately using 5/0 monofilament polypropylene sutures. Mice were placed on heating pads for recovery and then returned to the vivarium. The animals were monitored regularly to assess well-being and tumor recurrence. Mice were euthanized 28 days after primary tumor (PT) excision via isoflurane overdose, lungs were harvested, and metastases were enumerated.

#### 2.4.2 Stress paradigms

Prolonged alternating stress: In experiment 7, an alternating stress paradigm was employed, as described in the table below (Table 1), coupled with a twice-daily injection of vehicle to mimic drug injections in the next experiment (Exp. 8). All stress paradigms commenced during the light phase, starting 10 days after tumor inoculation.

**Table 1:** Experiment 7 – alternating stress paradigms.

	Stress Paradigm
Day (−3)	Morning: starting with 2 h restraint followed by 24 h tilt-light stress
Day (−2)	Morning: starting with 2 h wet cage. Evening: 12 h rat sawdust
Day (−1)	Morning: starting with 2 h restraint followed by 24 h food and water deprivation until surgery
Day (0)	Morning: Primary tumor removal followed by 24 h tilt-light stress
Day (+1)	Morning: 24 h rat sawdust
Day (+2)	Morning: starting with 2 h restraint followed by 24 h tilt-light stress

### 2.5 Experiment 8

#### 2.5.1 Cancer model

The 4T1.2 mammary carcinoma was inoculated as described in experiment 7. Resected tumors were placed in pre-weighed, sterilized Eppendorf tubes containing 300 μL RNAlater solution (Thermo Fisher) and stored in a −80 °C freezer until transcriptomic analysis.

#### 2.5.2 Stress paradigm

The stress paradigm was executed in the following schedule (Table 2), with or without pharmacological interventions. The stress paradigm was initiated 10 days after tumor inoculation, four days before primary tumor excision, and the termination of the study.

**Table 2:** Experiment 8 – alternating stress paradigms.

	Stress Paradigm
Day (−5)	Insertion of ALZET Osmotic Pumps 1 h before onset of dark phase
Day (−4)	Morning: starting with 2 h wet cage. Evening 2 h restraint
Day (−3)	Morning: Barn owl sound 24 h
Day (−2)	Morning: starting with 2 h restraint. Evening: 2 h wet cage
Day (−1)	Morning: Starting of 24 h tilt-light stress
Day (0)	Morning: Primary tumor removal

#### 2.5.3 Pharmacological intervention

As primary tumors drive the secretion of cancer-promoting inflammatory factors (Kartikasari et al., 2021), including in the 4T1 cell line (Benesch et al., 2015), the drug treatment in this study was designed to match our clinical study protocol (Shaashua et al., 2017), which includes propranolol + etodolac treatment in the perioperative context. This is despite the null effect of etodolac in the MADB106 model, which does not include surgical stress or primary tumor-related inflammatory processes.

Propranolol: All animals were surgically implanted (via a <1 cm incision made in the back of the neck) with Alzet® 7-day micro-osmotic pumps according to the manufacturer's protocol. Pumps were filled with either vehicle (PBS) or propranolol at a dose of 4 mg/kg/day.

Etodolac: Etodolac was dissolved in corn oil and administered once daily in a volume of 200 μL, starting on day 10 following tumor inoculation, at a dose of 10 mg/kg. Animals in the control group received 200 μL of vehicle.

#### 2.5.4 Transcriptomic analysis

##### 2.5.4.1 4T1 tumor gene expression profiling

Tumors were subjected to genome-wide transcriptional profiling conducted at the UCLA Social Genomics Core Laboratory as previously described (Haldar et al., 2023). Briefly, RNA was extracted from approximately 20 mg of frozen tumor tissue (Qiagen RNeasy), assessed for suitable mass (RiboGreen), reverse transcribed to cDNA using a high-efficiency mRNA-targeted enzyme system (Lexogen QuantSeq 3′ FWD), and subsequently sequenced using an Illumina NovaSeq instrument (Lexogen Services, GmbH). Sequencing targeted at least 5 million reads per sample (achieved mean = 7.8 million), each of which was mapped to the GRCm38 mouse reference transcriptome (average 94.6% mapping rate) and normalized to transcripts per million using the STAR aligner.

##### 2.5.4.2 Bioinformatics analysis

The differential expression of each gene was estimated with a standard linear statistical model relating log2-transformed transcript abundance values to main effects of Stress (vs control) and Drug (vs placebo), as well as a Stress × Drug interaction term to determine whether Drug treatment modified the effects of Stress on gene expression.

The hypothesis regarding EMT-related gene expression was analyzed using a previously established 130-gene signature designed to distinguish between epithelial-polarized and mesenchymal-polarized breast cancer cells (NCBI Gene Expression Omnibus GSE13915 (Choi et al., 2010)). Employing this predefined gene set, we examined if there was a notable difference in the average expression of the 67 mesenchymal-associated genes and the 63 epithelial-associated genes between groups.

To examine the a-priori hypothesis on key pro-inflammatory/anti-inflammatory (NF-κB, AP1, SP1, GR) transcription pathways, CREB family transcription factors, and hypoxic response regulators (HIF1, HIF2a), we applied the Transcription Element Listening System (TELiS, http://www.telis.ucla.edu/) bioinformatics analysis to all genes showing ≥1.5-fold differential expression as a function of the main effect of Stress (vs Control) and the Stress × Drug interaction term. These analyses test for differential prevalence of transcription factor-binding motifs within the core promoter DNA sequences of up-vs. down-regulated genes, using TRANSFAC position-specific weight matrices as previously described by Cole et al. (Cole et al., 2005, 2010). For all bioinformatics analyses, standard errors of the mean were computed and estimated by 200 bootstrap resampling cycles of linear model residual vectors across genes.

#### 2.5.5 Full list of stress paradigms

Tilt-light stress – as in Section 2.2.2.

Wet-cage: During the light phase, mice were placed in cages containing room-temperature water at a height of 1 cm for a total period of 2 h in an ambient temperature of 18 °C. Food and water were available *ad libitum*.

Restraint: Mice were placed in a transparent cylinder restrainer (50 ml volume Eppendorf conical tube) with ventilation holes from all sides. They were unable to move laterally or rotate in place for 2 h per session. The restrainers were placed in a fully lit room for the entire session. Food and water were unavailable during the session.

Rat sawdust: Mice were placed in a novel cage containing soiled rat sawdust, including urine and feces, for 12 h.

Food and water deprivation: Food and water were removed from the mice's home cages for a total duration of 22 h.

Barn owl sound: The experimental cages were placed in a separate room at 22 °C under a 12:12 light-dark cycle. A recording of a barn owl was constantly played for 24 h at 65 dB from speakers placed 50 cm from the cages.

### 2.6 Statistical analysis

All statistical analyses were conducted using JASP software (JASP Team, Netherlands, version 0.19.2, 2024) and GraphPad Prism (GraphPad Prism version 10.0.0 for Windows, GraphPad Software, Boston, Massachusetts, USA, www.graphpad.com). ANOVA and t-tests were conducted in line with the relevant assumptions, and Welch or Brown-Forsythe corrections were applied when appropriate (specific assumptions were not met). Post-hoc analyses were selected based on the primary analysis type. In experiments 4-6, we a priori defined two simple main effect tests for the drug in each of the stress levels. If ANOVA assumptions were violated, the Kruskal–Wallis tests were used. A deviation of more than 2 SD from the mean number of metastases was a priori considered a statistical outlier, and these subjects were reported and omitted from statistical analyses. All hypotheses were non-directional, and results with P < 0.05 were considered significant.

## 3 Results

### 3.1 Experiments 1-2

In the first two experiments, we tested the effects of the tilt-light stress paradigm on the number of metastases in models that involve a surgical procedure. Additionally, we examined whether there is a short period of stress, either before or after the surgery, that is sufficient to exert deleterious effects on metastatic development.

#### 3.1.1 Experiment 1 – tilt-light paradigm in the syngeneic MC38 cell line in C57BL/6J mice

**Fig. 1:**
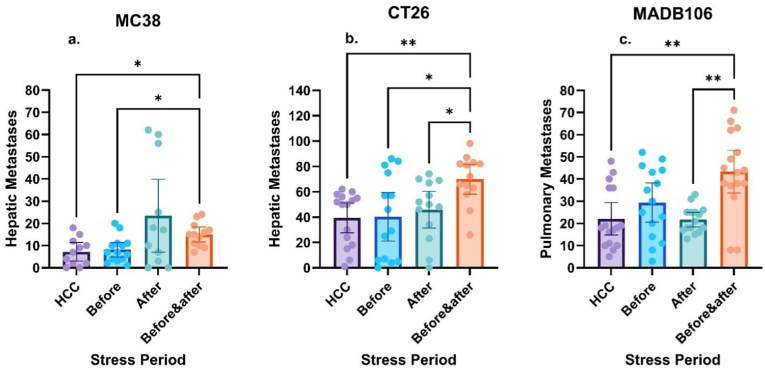
**Effects of psychological stress before and after tumor cell inoculation on metastases** Tilt-light stress for 48 h consistently increases the number of metastases across models. (a) In C57BL/6J mice inoculated with MC38 cells, before and after stress significantly elevated metastases compared to controls (p = 0.019) and compared to the before-inoculation stress group (p = 0.019). (b) In BALB/c mice injected with CT26 cells, before and after stress, significantly elevated metastases compared to controls (p = 0.003) and compared to the after-inoculation stress group (p = 0.023). (c) In the MADB106 model, before and after stress significantly increased pulmonary metastases compared with both control (p = 0.0043) and after-inoculation stress (p = 0.0012). Data represent mean ± 95 % CI. Brown-Forsythe ANOVA was used, followed by Games-Howell post hoc tests. ∗ (p < 0.05), ∗∗ (p < 0.01), and ∗∗∗ (p < 0.001). (For interpretation of the references to color in this figure legend, the reader is referred to the Web version of this article.)

Mice were subjected to a tilt-light stress paradigm for 24 h before or after cell inoculation, or at both time periods. Home cage control (HCC) animals stayed at the vivarium under standard housing conditions, except for tumor inoculation. Brown-Forsythe (B-F) ANOVA revealed a significant group difference in the number of hepatic metastases (F (3, 13.35) = 3.685, p = 0.0397) (Fig. 1a). Games-Howell post hoc test indicated that only the before and after stress group had a higher number of metastases compared with control animals (HCC; n = 11, M = 7.182; before and after; n = 12, M = 15, t (19.84) = 3.251, p = 0.019). The before and after group also showed a significant increase in metastases count compared with the group that endured stress only before tumor inoculation (n = 14, M = 8.143, t (23.799) = 3.187, p = 0.019).

#### 3.1.2 Experiment 2 – tilt-light paradigm using the syngeneic CT26 line in BALB/c mice

Mice were subjected to the same experimental paradigm as in experiment 1. B-F ANOVA showed a significant group difference (F (3, 42.62) = 4.412, p = 0.0087). Post-hoc analysis revealed that compared to the HCC group (n = 15, M = 39.333), the number of hepatic metastases in the before and after group was ∼1.75-fold higher (n = 13, M = 69.846, t (25.938) = 3.983, p = 0.003) (Fig. 1b). The before and after group was also significantly higher than the group enduring stress only before or only after the inoculation (n = 14, M = 40.143, t (21.141) = 2.871, p = 0.042; n = 14, M = 42.571; t (23.84) = 3.106, p = 0.023).

#### 3.1.3 Experiment 3 – tilt-light paradigm in the syngeneic MADB106 cell line in F344 rats

In this experiment, we proceeded to examine the effects of stress on metastatic development in a model that does not entail surgical stress. In this manner, we could examine the effects of psychological stress and its blockade in isolation from surgical-physiological stress.

The same design and tilt-light paradigm as described in experiments 1-2 were employed in F344 rats. 64 female and male rats were randomly assigned to the 4 experimental groups. Due to outlying values, two male subjects were removed (one from the group undergoing stress 24 h before the inoculation and one from the group that experienced stress only after the inoculation). Preliminary ANOVA revealed no effect for sex (p = 0.375), nor an interaction of stress with sex (p = 0.322), hence, sex was not used as an independent factor. Consistent with the previous experiments, B-F ANOVA showed significant group differences (F (3, 45.71) = 8.108, p < 0.001). According to post-hoc analysis, the before and after stress condition had a significantly higher number of pulmonary metastases (n = 16, M = 43.375), compared to the HCC (n = 16, t (28.042) = 3.748, M = 22.125, p = 0.0043) and the after condition (n = 15, t (18.366) = 4.563, M = 21.667, p = 0.0012) (Fig. 1c).

### 3.2 Experiments 4-6

As the before and after stress condition showed the most consistent results, the next 3 studies employed this paradigm and focused on blocking the deleterious effects of stress on metastases pharmacologically. F344 rats were used to study the effects of glucocorticoid, β-adrenergic, or prostaglandin signaling in inducing a stress-dependent increase in pulmonary metastases by employing RU-486, propranolol, or etodolac, respectively. In all experiments, the stress paradigm resulted in statistically significant weight loss, a common response in rodent models of acute stress (Harris, 2015), providing an additional validation for the stress paradigm's effectiveness regardless of treatment used (see supplementary material A1).

#### 3.2.1 Experiment 4 – the effects of RU-486, a glucocorticoid antagonist, on metastatic development under stress

Fifty-nine male F344 rats were divided into 4 groups – HCC-vehicle, HCC-RU-486, stress-vehicle and stress-RU-486. Due to outlying values, three animals were excluded from the statistical analysis (two from HCC-RU-486, and one from stress-vehicle), eventually yielding 11-16 animals per group. The study was conducted in males to reduce the potential confounding from blocking progesterone receptors with RU-486, as the development of MADB106 metastases is ovarian hormone-sensitive (Ben-Eliyahu et al., 1996).

**Fig. 2:**
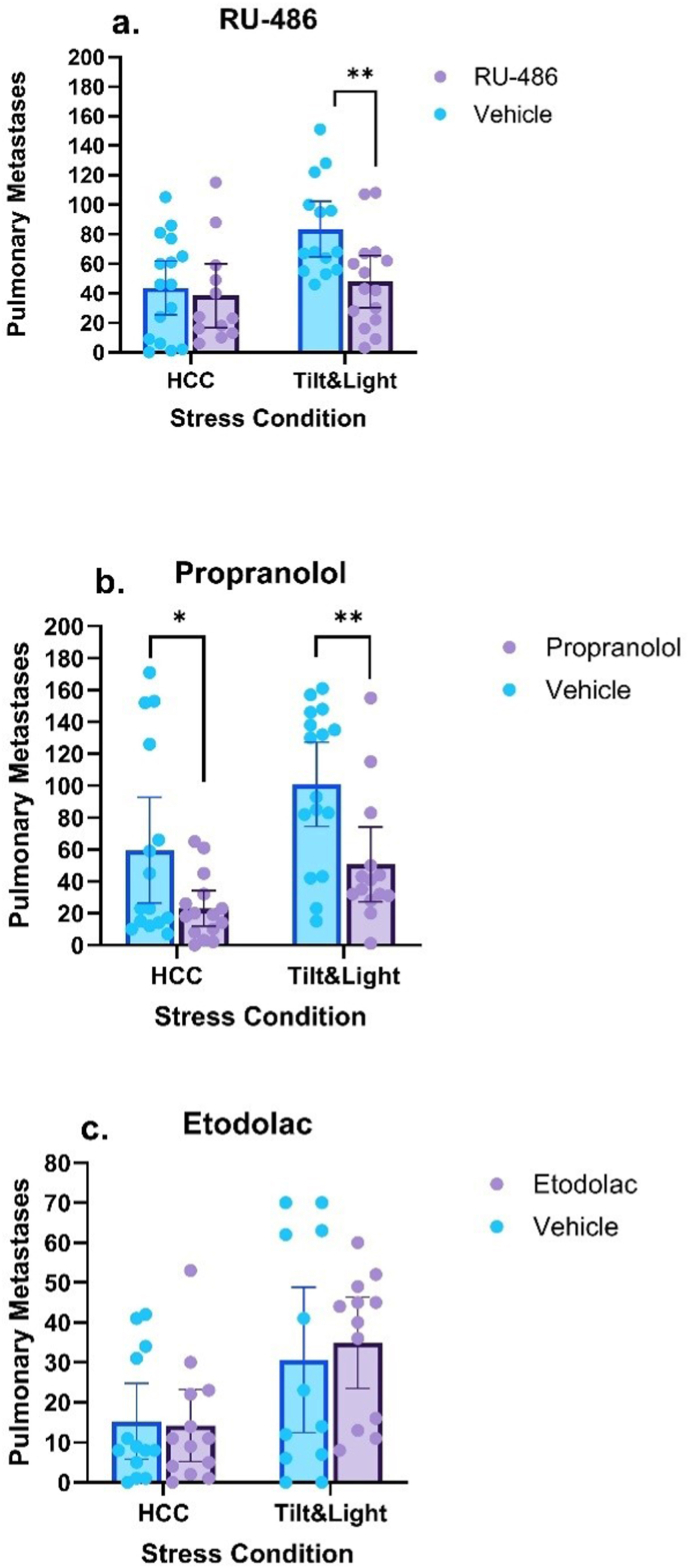
**Pharmacological blockade of perioperative psychological stress** Pharmacological blockade of 48-h tilt-light stress in MADB106 pulmonary metastases. In all three studies (a, b, and c), stress increased the number of metastases (*p <* 0.005). (a) RU-486 abolished the effect of stress, reducing metastasis counts to baseline levels in the stress groups (*p =* 0.004). (b) Propranolol significantly reduced metastases in both control and stress conditions (*p =* 0.031 and *p =* 0.008). (c) Etodolac did not affect metastatic count (*p =* 0.437) in any of the stress levels. Data represent mean ± 95 % CI. ∗ (p < 0.05), ∗∗ (p < 0.01), and ∗∗∗ (p < 0.001).

Preliminary analysis indicated a strong correlation between the number of metastases and the animal's weight (n = 56, r = −0.672, p < 0.001), hence, ANCOVA was used. According to this analysis, there was a significant main effect for stress (F (1, 51) = 10.673, p = 0.002) with an interaction between stress and RU-486 treatment (F (1, 51) = 5.934, p = 0.018). Simple main effects analysis of RU-486 indicated that the drug decreased the number of metastases only under the stress condition (p = 0.004) (Fig. 2a).

#### 3.2.2 Experiment 5 – the effects of propranolol on metastatic development under stress

This experiment employed the same design as experiment 4, testing the ability of propranolol to reduce the effects of stress. Sixty-three male and female F344 rats were used. Three rats (HCC-propranolol, stress-propranolol, stress-vehicle) were removed from the analysis due to being statistical outliers, and an additional one was excluded due to unsuccessful tumor cell injection (stress-propranolol). No significant sex effect or interaction with sex was observed, and data from both sexes were analyzed together. Two main effects were observed, for the stress condition and the drug treatment. Tilt-light stress for 48 h significantly increased the number of metastases by nearly twofold, to an average of 80.069 metastases (n = 29), compared to HCC (n = 30, H (1) = 12.221, p < 0.001). Propranolol treatment (n = 28) also had an effect of a similar magnitude, reducing the average number of metastases to 37.679, compared with M = 80.839 in the control group (H (1) = 6.742, p = 0.009). Simple main effects analysis of propranolol indicated that the drug decreased the number of metastases under both HCC (p = 0.031) and the stress condition (p = 0.008) (Fig. 2b).

#### 3.2.3 Experiment 6 – the effects of etodolac on metastatic development under stress

The study was conducted in 53 females. In the statistical analysis, three animals were excluded (HCC-etodolac, HCC-vehicle, stress-etodolac) due to being outliers, and for statistical analyses, each group had 12-13 animals. Similar to the previous two experiments, the tilt-light paradigm led to a significant increase in the pulmonary metastatic load (H (1) = 8.22, p = 0.004), doubling their average from 14.769 metastases in the HCC group (n = 26), to 32.792 metastases in the stress group (Fig. 2c). Nevertheless, etodolac treatment did not have any effect on the number of metastases (H (1) = 0.604, p = 0.437), and none of the simple main effects of the drugs were significant (p > 0.6).

### 3.3 Experiments 7-8

#### 3.3.1 Experiment 7: the effects of prolonged perioperative stress on metastatic load in spontaneously metastasizing mammary carcinoma modelling breast cancer

Next, we wanted to explore the effect of psychological stress in the perioperative period on the development of metastases, in a manner that better mimics the clinical setting, where the metastatic process and the psychological stress are simultaneously ongoing for several days during the perioperative context of primary tumor excision. In this experiment, female BALB/c mice were divided into stress versus HCC groups (n = 10/group). Mice were inoculated with the 4T1 mammary cancer cell line into the mammary fat pad (1 × 10^5^ cells/animal), and the developing PTs were removed with a minimal surgical procedure 14 days post inoculation. Animals in the stress group underwent a 6-day alternating stress paradigm. The alternating stress paradigm began three days before tumor resection and continued for 48 h, as described in the Methods section (see Table 1). In addition, all animals received vehicle injections twice daily, to be included as a treatment regimen in the following experiment and to partially simulate medical perioperative procedures. Mice were euthanized 28 days after PT excision, and lung metastases were enumerated.

**Fig. 3:**
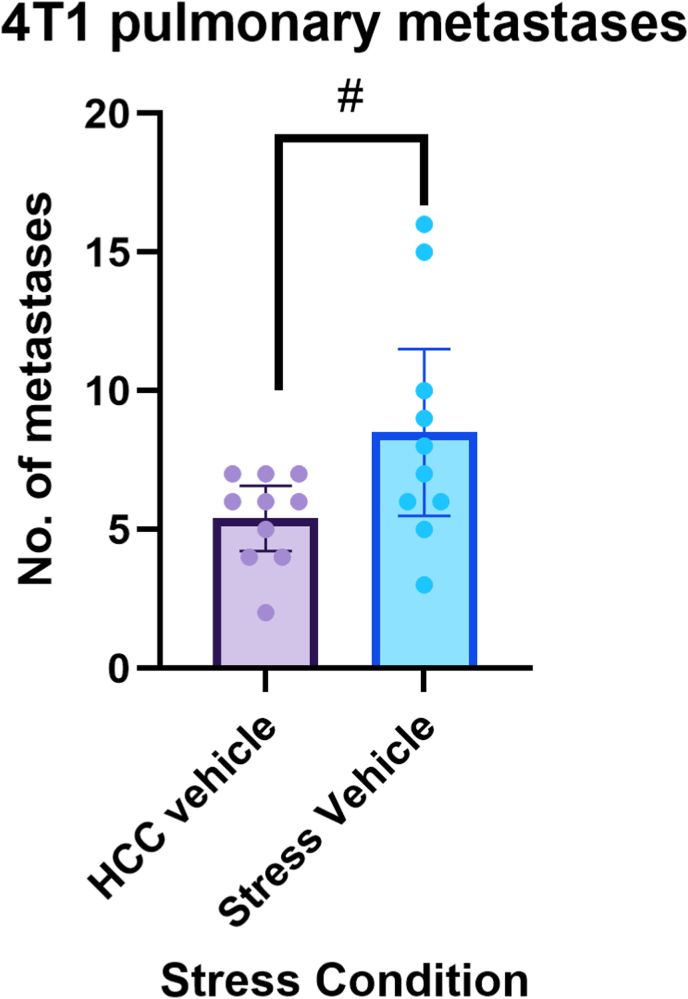
Effects of perioperative alternating psychological stress on spontaneous metastases Six perioperative days of alternating stress increase pulmonary metastasis in the 4T1 mammary carcinoma spontaneously metastasizing model. Orthotopic primary tumors were removed surgically on day 14 post-tumor inoculation. Four weeks following tumor excision mice exposed to perioperative stress developed nearly twice the number of pulmonary metastases compared to HCC group (*p =* 0.0509). Data represent mean ± 95 % CI. # (p < 0.09) Data represent mean ± 95 % CI. The Welch *t*-test was used.

Results indicated that mice exposed to perioperative stress developed more than 1.5-fold pulmonary metastases (M = 8.5) compared to their HCC counterparts (M = 5.4, Welch *t*-test (11.707) = −2.175, p = 0.0509) (Fig. 3).

#### 3.3.2 Experiment 8: the effects of 4 pre-operative days of stress, and of pharmacological blockade of stress-inflammatory signaling on 4T1 primary tumor transcriptomic profiling

To study the effect of pre-operative stress exposure and of pharmacological blockade of stress-inflammatory signaling on tumor transcriptome, we employed a 2X 2 design: Stress Vs. HCC by propranolol + etodolac Vs. vehicle treatment, N = 8/group. Primary 4T1 tumors were inoculated in 32 female BALB/c mice. On day 10 post tumor inoculation, stress exposure commenced, and on day 14, all tumors were removed immediately following isoflurane anesthesia, tumor RNA was extracted, sequenced, and genome-wide transcriptional profiling of the excised 4T1 PTs was conducted.

**Fig. 4:**
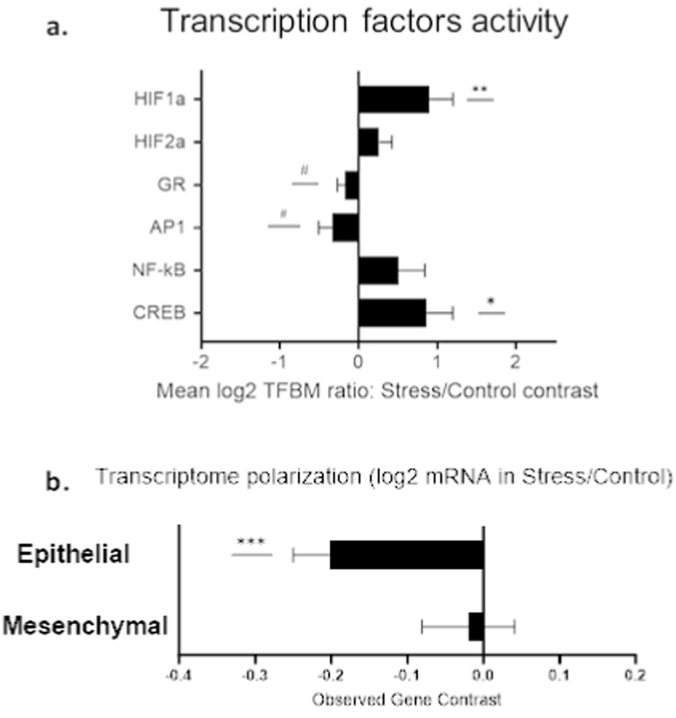
The effect of pre-operative alternating stress on tumor transcriptional activity The effect of stress on tumor transcriptome in Stress-vehicle vs. Control-vehicle groups**. a.** TELiS promoter-based bioinformatics analysis of transcription factor–binding site prevalence in promoters of gene transcripts from all genes that showed ≥1.5-fold difference. **b.** Differential expression of log2 mRNA from epithelial and mesenchymal gene sets. ± SEM. n = 8 per experimental group. # (p < 0.09) ∗ (p < 0.05), ∗∗ (p < 0.01), and ∗∗∗ (p < 0.001).

The effects of stress: In the stress-vehicle vs the control-vehicle group comparison, primary analysis identified 108 genes showing >1.5-fold upregulation, and 313 genes showing >1.5-fold downregulation. TELiS analysis of a priori hypothesized transcription factor binding motif prevalence in the promoters of differentially expressed genes indicated stress-induced activation of the HIF transcription factor involved in hypoxia signaling (V$HIF1_Q3, 0.893 ± 0.307, p = 0.004) and CREB family transcription factors involved in neural/adrenergic signaling (V$CREB_02, 0.864 ± 0.334, p = 0.01). However, results showed no significant differential activity of AP1 (V$AP1_Q6_01, −0.332 ± 0.174, p = 0.058), NF-kB (V$NFKB_Q6_01, 0.509 ± 0.331, p = 0.155), glucocorticoid receptor (V$GR_Q6_01, −0.175 ± 0.094, p = 0.06) or HIF2a transcription factor binding motifs (TFBMs), (V$HIF2A_01, 0.261 ± 0.161, p = 0.1) (Fig. 4a). To examine the effect of stress on epithelial-to-mesenchymal transition (EMT), we tested differences in average expression of 67 mesenchymal-characteristic genes and 63 epithelial-characteristic genes defined in previous research (Choi et al., 2010; Lutgendorf et al., 2018). Tumors from stressed mice showed a significantly decreased expression of the epithelial gene composite (−0.200 ± 0.049, p = 0.0001) but no difference in the mesenchymal gene composite (−0.0201 ± 0.061, p = 0.743) (Fig. 4b). These results indicate a higher EMT profile through lower epithelial gene expression, which is considered a pro-metastatic biomarker (Ribatti et al., 2020).

##### 3.3.2.1 The effect of stress × drugs interaction on tumor transcriptome

To determine whether propranolol + etodolac treatment (a β-adrenergic blocker and a COX-2 inhibitor, respectively) modified the effects of stress exposure on tumor transcriptome, we identified all genes showing ≥1.5-fold change in Stress × Drug interaction analysis (534 upregulated genes and 357 downregulated).

**Fig. 5:**
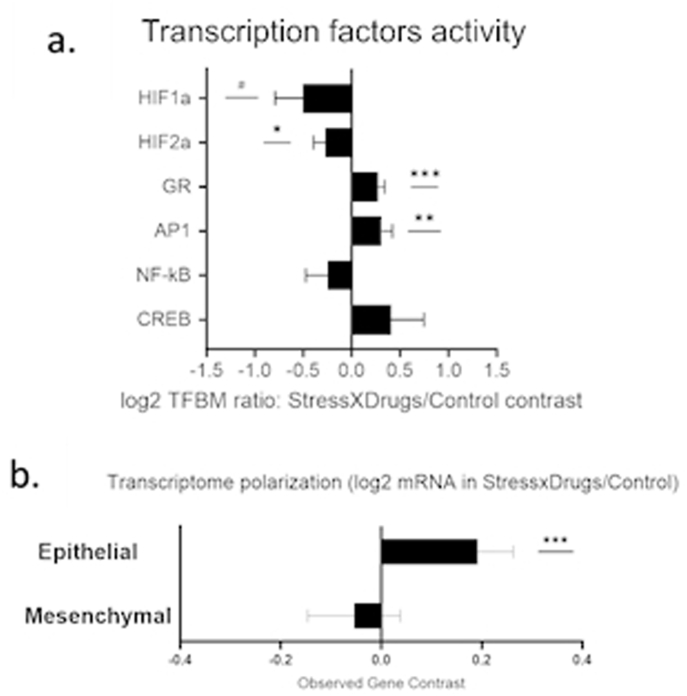
The effects of drug treatment and pre-operative stress on tumor transcriptomic activity The effect of the Stress × Drug interaction vs. Control. a. TELiS promoter-based bioinformatics analysis of transcription factor–binding site prevalence in promoters of gene transcripts from all genes that showed ≥1.5-fold difference. b. Differential expression of log2 mRNA from epithelial and mesenchymal gene sets. ± SEM. n = 8 per experimental group. # (p < 0.09) ∗ (p < 0.05), ∗∗ (p < 0.01), and ∗∗∗ (p < 0.001). Drugs – propranolol + etodolac.

TELiS analysis of this interaction indicated that Drug treatment significantly enhanced the effects of Stress on the activity of the glucocorticoid receptor (V$GR_Q6_01, 0.273 ± 0.068, p = 0.0001) and the pro-inflammatory AP1 transcription factor (V$AP1_Q6_01, 0.309 ± 0.111, p = 0.005). Drug treatment significantly reduced the effect of Stress on activity of the HIF2a transcription factor (V$HIF2A_01, −0.272 ± 0.122, p = 0.027), and showed no significant modification of NF-KB, HIF1, or CREB TFBMs (V$NFKB_Q6_01, −0.243 ± 0.230, V$HIF1_Q3, -0.505 ± 0.285, p = 0.07, V$CREB_02, 0.408 ± 0.339, p = 0.23) (Fig. 5a).

To directly examine effects on EMT, we tested for Stress × Drug interactions in the expression of epithelial and mesenchymal gene composites as described above. Drug treatment significantly reversed the stress-induced reduction in epithelial gene expression (0.192 ± 0.069, p = 0.007), while not affecting the mesenchymal gene composite (−0.054 ± 0.092, p = 0.55) (Fig. 5b). The relative change in the epithelial composite was similar in magnitude and in opposite direction to the basic effect of stress in the absence of drug treatment, suggesting that drug treatment can successfully block the effects of stress on pro-metastatic biology in this paradigm.

## 4 Discussion

The current study explored the unique impact of psychological stress by combining elements of discomfort, environmental change, and fear (Jaggi et al., 2011; Atrooz et al., 2021), in facilitating metastatic development, both in the context of surgery and in its absence, using the tilt-light stress paradigm. We further examined the underlying mechanisms of the effects of stress, both at the neuroendocrine level as well as at the molecular level of the developing primary tumor.

The tilt-light paradigm, which we have previously shown to increase plasma corticosterone levels (Matzner et al., 2019), consistently elevated the number of metastases in all models tested (CT26, MC38, and MADB106), regardless of surgical procedure. When the tilt-light paradigm was employed for only a 24-h period, before or after tumor inoculation, the effects were small and inconsistent, but when employed for the entire 48-h surrounding tumor cell inoculation, the effects were robust. While it is not clear whether the extended duration, or the combined pre- and post-exposure, is key to the robustness of the 48-h impact, the clinical setting is clearly characterized by both elements and for longer periods. It is important to note that while the tilt-light paradigm induces a substantial physiological stress response, as reflected by an increase in plasma corticosterone (Matzner et al., 2019), reduced body weight, and an increase in metastases, we did not test its effects on long-term changes in anxiety- and depressive-like behavior. Such long-term effects could further dampen anti-cancer immunity and drive cancer progression (Dhabhar et al., 2012). Therefore, addressing these variables may be highly relevant in identifying individual differences in cancer susceptibility and progression, and could be assessed by future work. Overall, perioperative psychological stress can significantly affect long-term cancer outcomes independently of the impact of surgery itself in rodents, as was also suggested by previous studies (Matzner et al., 2019) and reviews, including in humans (Hanalis-Miller et al., 2022; Sandbank et al., 2023). Markedly, the effect size of ∼2-fold increase in number of metastases in the 48-h stress paradigm, is equivalent to the effect size induced by acute administration of 0.1 mg/kg LPS, or half the effect size of laparotomy (4 cm mid-abdominal incision), employing the same MADB106 tumor model and outcome (see supplementary A2-A3) and (Melamed et al., 2005; Naor et al., 2009).

To pharmacologically counteract the effects of the tilt-light stress in the MADB106 experimental lung metastases model that does not involve surgery, we used RU-486 (mifepristone), a glucocorticoid antagonist and antiprogestogen; propranolol, a non-selective β-adrenergic blocker; and etodolac, a COX-2 inhibitor. Propranolol and mifepristone, but not etodolac, each completely reversed the metastasis-promoting effects of stress, implying the involvement of both β-adrenergic and glucocorticoid signaling. It also suggests that there is some synergism between these signaling pathways in this setting, as blocking each of them alone was sufficient to nullify the effects of stress.

Glucocorticoid receptors are widely expressed in immunocytes (Baschant and Tuckermann, 2010), and mifepristone was previously shown to block surgery- and stress-induced suppression of NK cell activity and consequent increase in MADB106 metastases (Rosenne et al., 2014). Mifepristone was also shown to hinder the progression of various cancer types, including breast (Gaddy et al., 2004), glioma (Ramaswamy et al., 2012), prostate (Tieszen et al., 2011), and ovarian cancers (Goyeneche et al., 2007). Additionally, mifepristone has been shown to modulate cancer-related intracellular processes, including YAP (Puhr et al., 2018) and Cdk2 activity (Tieszen et al., 2011; Puhr et al., 2018). Thus, in our current study, mifepristone may have acted through several mechanisms by affecting immune activity and malignant tissue. The specific immune and tumor characteristics affected by mifepristone treatment should be studied in the future to gain insights into potential clinical implementation of this drug or alternative approaches. Furthermore, in the current study, we have studied mifepristone's effects only in male rats to minimize possible interaction with sex hormones, as mifepristone is a potent antiprogestogen (Sitruk-Ware and Spitz, 2003), yet a limited off-target effect cannot be fully ruled out. To facilitate better generalizability of the outcomes to females, future investigation would have to assess the drug's interaction with progesterone under perioperative stress conditions. Interestingly, mifepristone was tested pre-clinically and clinically as a therapeutic agent for mood and anxiety disorders (e.g., depression and PTSD), but yielded inconsistent effects (Gallagher and Young, 2006; Ding et al., 2021; Nayana et al., 2022; Golier et al., 2023; Yalin et al., 2023). Furthermore, sustainable pharmacological blockade of glucocorticoids to address mood disorders is often not achieved due to compensatory mechanisms (Milo et al., 2025). Moreover, it is clinically challenging to perioperatively limit steroid levels, which rise naturally during surgery, limit inflammation (Coutinho and Chapman, 2011), and are often administered to reduce side effects of cancer treatments (Prete et al., 2018; Kim et al., 2023). Consequently, herein and in our clinical studies, we have focused on the use of propranolol, with and without etodolac, to pharmacologically address deleterious perioperative stress-inflammatory responses.

Propranolol, used in the tilt-light paradigm, also significantly reduced the number of MADB106 pulmonary metastases. In this tumor model, the β-adrenergic signaling was previously shown to suppress NK activity and consequently increase metastasis (Shakhar and Ben-Eliyahu, 1998; Melamed et al., 2005). Additionally, propranolol was repeatedly shown to attenuate cancer progression, and β-adrenergic activation in the tumor microenvironment was shown to inhibit anti-cancer immunity and directly facilitate malignant tissue growth and metastasis (Eng et al., 2014; Mravec et al., 2020). In our previous studies, both propranolol and its isomer nadolol, which does not cross the blood-brain barrier, were similarly effective in blocking the impact of surgical stress (Melamed et al., 2005; Benish et al., 2008), suggesting a significant peripheral effect of propranolol in the context of surgery. However, in the current study, which employed a 48-h psychological stress paradigm, the protective effects of propranolol may have also been related to its central nervous system anxiolytic effects (Steenen et al., 2016). The off-label use of propranolol to combat stress-related psychiatric disorders is well documented, indicating its capacity to modulate central sympathetic signaling, reducing anxiety-associated physiological symptoms, and possibly assisting in inhibiting the reconsolidation of fear memories (Steenen et al., 2016; Deng et al., 2020). Last, the clinical use of propranolol in cancer patients (Jang et al., 2017; Ramondetta et al., 2019; Hiller et al., 2020; Knight et al., 2020), and in conjunction with etodolac in our randomized-controlled trials during the perioperative period (Shaashua et al., 2017; Haldar et al., 2020), has shown positive effects on excised tumor biomarkers (EMT and metastasis-linked transcriptional activity), immunocyte tumor infiltration, markers of cancer progression, and long-term disease outcomes (i.e., disease-free survival) (Shaashua et al., 2017; Haldar et al., 2018, 2020; Ricon-Becker et al., 2023a; Hiller et al., 2020; Knight et al., 2020; Ramondetta et al., 2019; Jang et al., 2017; Hüttner et al., 2025; Carnet Le Provost et al., 2023).

The COX-2 inhibitor, etodolac, did not improve the effects of the tilt-light stress paradigm on metastatic outcomes in the MADB106 model, suggesting a limited role of inflammatory prostaglandins in the context of this 48-h stress paradigm in rats. In the context of surgery, however, COX inhibitors alone, including etodolac, were effective in reducing the metastasis-promoting effects of surgery in our previous translational studies in this model (Yakar et al., 2003; Benish et al., 2008), suggesting the contribution of surgery-induced tissue damage and consequent inflammation to the promotion of metastasis (Horowitz et al., 2015; Hiller et al., 2018; Eckerling et al., 2021). Additionally, in our clinical trials in breast cancer patients, we observed a pre-operative circulation increase in the inflammation markers IL-6 and CRP, which were completely abrogated by 5 pre-operative treatment days with etodolac + propranolol (Shaashua et al., 2017). In a small clinical trial in ovarian cancer patients, propranolol treatment alone did not significantly affect perioperative CRP levels, while positively affecting the tumor marker CA-125 (Jang et al., 2017). Together, these translational and clinical studies suggest that COX-2 inhibition may be important not only in the context of surgery (Benish et al., 2008; Glasner et al., 2010) and inflammation-inducing malignancy treatments (e.g., chemotherapy), but also in the context of psychological stress in conjunction with propranolol, especially before the malignant tissue has been removed.

Unlike the clinical context in which we cannot disentangle perioperative psychological from physiological stress in their impact on growing tumor tissue, herein we used a model of a spontaneously metastasizing mammary cancer, where we employed propranolol + etodolac treatment in conjunction with psychological stress to study tumor transcriptomic alterations after 4 days of stress exposure before tumor excision. While tumors were removed surgically in this model, the excision procedure was brief, lasting approximately 2 minutes until full tumor removal and preservation, with tissue damage being limited to a localized skin segment surrounding the subcutaneous tumor. Although we cannot completely rule out some involvement of tissue damage on transcriptomic effects, the short period of 2 minutes is believed to be insufficient in altering the studied transcriptomic indices, which were also halted by the preservation procedure (Molina et al., 2013; Meeussen and Lenstra, 2024). Thus, we believe that psychological stress is the key contributor to these transcriptomic outcomes.

Specifically, pre-operative stress downregulated the expression of epithelial composite genes, indicating partial EMT, which was reversed by the drug treatment. The process of EMT is sensitive to both adrenergic activity (Silva et al., 2022) and COX-dependent inflammatory responses (Gómez-Valenzuela et al., 2021), and is characterized by loss of cell-cell adhesion, increased motility and invasiveness, and pro-metastatic activity, including immune resistance, as well as resistance to apoptosis (Ribatti et al., 2020; Kang et al., 2021; Imodoye and Adedokun, 2023). Additionally, stress significantly upregulated the activity of HIF1-a TF, which is indicative of adaptation to a hypoxic environment, and linked to increased metastatic potential and resistance to anti-cancer treatment (Semenza, 2003). The drug treatment in the stress group downregulated HIF activity, with a marginally significant decrease in HIF-1a and a significant decrease in HIF-2a, another member of the HIF TF family. Moreover, we have observed a marginally significant decrease in AP1 activity in the stress condition, and a significant upregulation under stress with drug treatment. While AP1 is often considered an oncogenic complex, it also exhibits anti-cancer functions by inducing apoptosis and suppressing tumor formation under certain conditions (Eferl and Wagner, 2003; Hess et al., 2004). A similar pattern of effects was observed in the transcriptional activity of the glucocorticoid receptor (GR). GRs are commonly expressed in many types of solid tumors (Block et al., 2017), and while increased glucocorticoid receptor expression and activity are markers of negative prognosis (Volden and Conzen, 2013), in some cancer types GR expression shows opposite indications. For example, in a meta-analysis conducted by West et al., higher expression of GR was in fact correlated with improved DFS outcomes (West et al., 2016). Similarly, in a study on patients with adrenocortical carcinoma, higher GR was associated with a better tumor immune status and disease trajectories, along with increased survival rates (Wu et al., 2022). Hence, the interpretation of GR TF activity should be carefully considered (Khadka et al., 2023). Lastly, stress exposure increased CREB activity in the tumor tissue in a significant manner, while under drug treatment, this increase lost statistical significance. Activation of β-adrenergic receptors by catecholamines is known to lead to a cascade of responses leading to enhanced expression of CREB TFs, which in turn promote an inflammatory state and support cancer proliferation, angiogenesis, and invasion (Nilsson et al., 2020). Taken together, these findings indicate that pre-operative stress alone can markedly promote several known biomarkers of metastatic progression in the primary tumor, while the drug treatment of propranolol + etodolac can reverse or attenuate these effects. Future studies should examine whether the impact of this treatment stems from its direct modification of the tumor and its microenvironment, or through broader interactions with the immune and nervous system, as well as the prognostic value of the observed transcriptional changes.

Given that psychological stress alone may have a long-term impact on cancer outcomes, the perioperative timeframe presents a crucial opportunity for various interventions to optimize disease course (Ben-Eliyahu, 2020). Intervention may include psycho-behavioral approaches (Chang et al., 2022) along pharmacological means to reduce surgery-related stress and inflammatory responses (Ricon-Becker et al., 2023b). In fact, in a recent clinical trial in breast cancer patients, we showed that a preoperative psycho-behavioral intervention alone improved biomarkers of metastasis in excised tumors (Hanalis-Miller et al., 2024). Additionally, some perioperative approaches have focused on holistic pre-habilitation protocols, entailing a combination of physical, psychological, and nutritional aspects, which are complementary to each other (Trépanier et al., 2019; Santa Mina et al., 2021; Antoni et al., 2023). Indeed, pooled data from three recent prospective studies suggest that such a multimodal approach could improve disease-free survival for stage III colorectal cancer patients (Trépanier et al., 2019). A recent study by Sabajo et al. also showed that pre-habilitation reduced the risk for complications, while being cost-effective despite extensive pre-surgical investment (Sabajo et al., 2024). Yet, additional research into optimal execution of such perioperative treatments and their long-term cancer efficacy is necessary (Antoni et al., 2023).

Despite the use of four tumor models, this study has several limitations. First, while the tilt-light paradigm mimics different distressing aspects of the perioperative period, such as novel and spatially limited environment, altered sleep and feeding, ambient discomfort, and more, its potential specific physiological perturbations, such as chrono-disruption (Savvidis et al., 2024) or mild hypothermia (Sandbank et al., 2024), may contribute to metastatic development. Future studies may investigate these aspects in-depth. Additionally, while significant reductions of metastatic load using a **β**-adrenergic blocker and an antiglucocorticoid were observed, it remains unclear to what degree these protective effects are mediated via direct action on tumor tissue, immune modulation, or central nervous system pathways. A similar limitation exists regarding the mammary primary tumor transcriptional changes, where further mechanistic work is needed to identify the source of the effect and a potential direct causal link between the observed changes and long-term immune and/or cancer outcomes.

Taken together, our results indicate that psychological distress on its own, in the context of significant or minimal surgical assault, can markedly enhance the perpetuation of metastatic processes independently of the impact of surgery. These findings, and the promising effects of diverse perioperative clinical interventions, call for a comprehensive and personally tailored stress-management care, including psycho-behavioral approaches, in patients diagnosed with solid cancers, particularly during the critical perioperative period.

## CRediT authorship contribution statement

**Bar Bruno Shvalbo:** Conceptualization, Data curation, Formal analysis, Investigation, Writing – original draft. **Sharon Scarlat:** Conceptualization, Data curation, Investigation. **Nahida Sakis:** Investigation, Writing – review & editing. **Estherina Trachtenberg:** Formal analysis, Investigation, Writing – original draft. **Elad Sandbank:** Investigation, Methodology. **Meshi Weil:** Investigation. **Anabel Eckerling:** Investigation, Writing – review & editing. **Steven W. Cole:** Formal analysis, Methodology. **Shamgar Ben-Eliyahu:** Conceptualization, Funding acquisition, Supervision, Writing – original draft.

## Funding and support

This work was supported by the Israeli Science Foundation (ISF), the Israeli Ministry of Science, the Israeli Ministry of Health, and the Cancer Biology Research Center (CBRC) at Tel Aviv University.

## Declaration of competing interest

The authors declare that they have no known competing financial interests or personal relationships that could have appeared to influence the work reported in this paper.

## Data Availability

Data will be made available on request.
