# Supplementary material for: Perioperative psycho-behavioral stress promotes cancer metastasis beyond the impact of surgery: Neuroendocrine and tumor molecular mediating mechanisms
Source: Brain Behav Immun Health. 2026 Mar 26;54:101228. doi: 10.1016/j.bbih.2026.101228 (PMC13091779; doi:10.1016/j.bbih.2026.101228)
Supplement: Multimedia component 1 [file mmc1.docx]

Supplementary material A

1 – The effect of the tilt-light stress on weight in F344 rats.


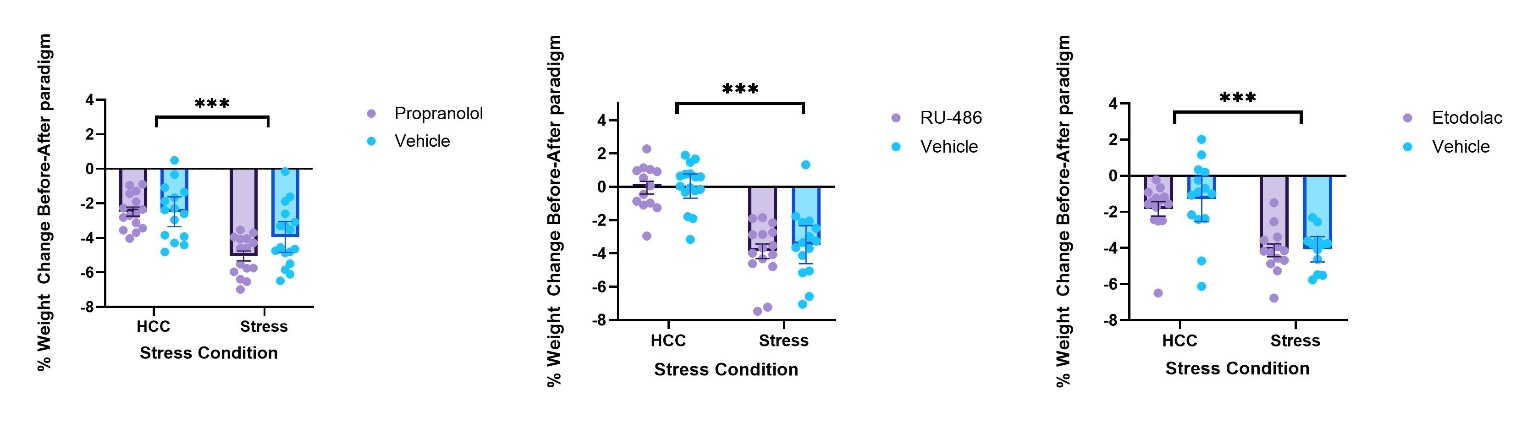
In experiments 4-6, the relative weight change after the stress paradigm was used as a validation for its effectiveness. In all of these experiments, the tilt and light stress led to a significant weight loss (p < 0.001), with no significant effect for the drugs or for the interaction of stress with the drugs. No animals were removed from this analysis except for one subject with a missing datapoint in the HCC-Propranolol group.

In all three pharmacological studies employing the tilt-light stress paradigm surrounding MADB106 tumor inoculation, significant weight loss (p < 0.001) was observed in the stress-exposed group compared to HCC. Error bars represent SEM.

2 – The effects of LPS on metastatic development in F344 rats.

Methods:

Rats: Eight to nine months old female F344 rats (n=10-11 per group) were randomly assigned to one of the experimental groups. Animals were maintained and monitored as described in the paper’s Methods section.

Cancer model: The MADB106 mammary adenocarcinoma was inoculated into the tail vein of 21 female F344 rats, after cell preparation as described in the main body of the paper. One animal (vehicle group) was removed as a statistical outlier. Animals were euthanized, and lungs were harvested for metastases enumeration 17 days after inoculation, once signs of illness appeared.

LPS administration: Lipopolysaccharide derived from Escherichia coli (Sigma, Israel) was diluted in PBS and administered i.p. at 0.1 mg/kg upon tumor inoculation under isoflurane anesthesia (Naor, 2009). Beforehand, while awake, each animal received a vehicle injection to mimic the rest of the experimental conditions.

Results:

LPS administration led to a significant ~2.5-fold increase in lung metastases compared to the vehicle group (LPS: n = 10, M = 108.7; vehicle n=10, M=43.1; t(18) = 3.063, p = 0.007).


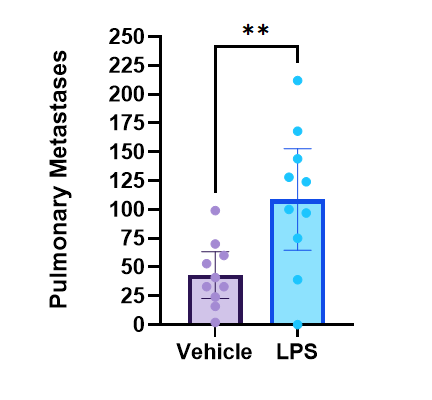


A single LPS administration led to an approximately 2.5-fold increase in the number of pulmonary metastases (t(18) = 3.063, p = 0.007). Error bars represent 95% CI.

3 – The effects of laparotomy on metastatic development in F344 rats.

Methods:
Rats: Six-month-old female F344 rats were randomly assigned to the laparotomy (n=13) or HCC (n=15) group. Animals were maintained and monitored as described in the paper’s Methods section (IACUC approval number: TAU - LS - IL - 2308 - 152 – 5).

Cancer model: The MADB106 cancer model was used as described in the previous section.

Experimental laparotomy: Animals were anesthetized under 2.5% volatile isoflurane. A four-centimetre midline abdominal incision was made, and the small intestines were externalized. The small intestines were rubbed with PBS-soaked gauze and left hydrated for 20 minutes. Finally, the intestines were internalized, the incision was sutured with 3/0 non-absorbable polyamide monofilament, cleaned, and treated with lidocaine. The procedure was carried out two hours before cancer cell inoculation.

Enumeration of pulmonary metastases: Rats were monitored daily for general well-being. Once signs of sickness appeared on post-inoculation day 21, rats were euthanized with an isoflurane overdose, and lungs were harvested. The tissue was preserved in Bouin’s solution. Metastases were enumerated by an experimenter blind to the experimental conditions.

Results:

Two animals were removed from analysis due to deviation of more than 2-SDs from the mean (one from each group). Rats that had undergone laparotomy exhibited a significant (Welch’s t(11.719) = 3.931, p < 0.002) ~four-fold increase in the number of pulmonary metastases compared to the HCC group.


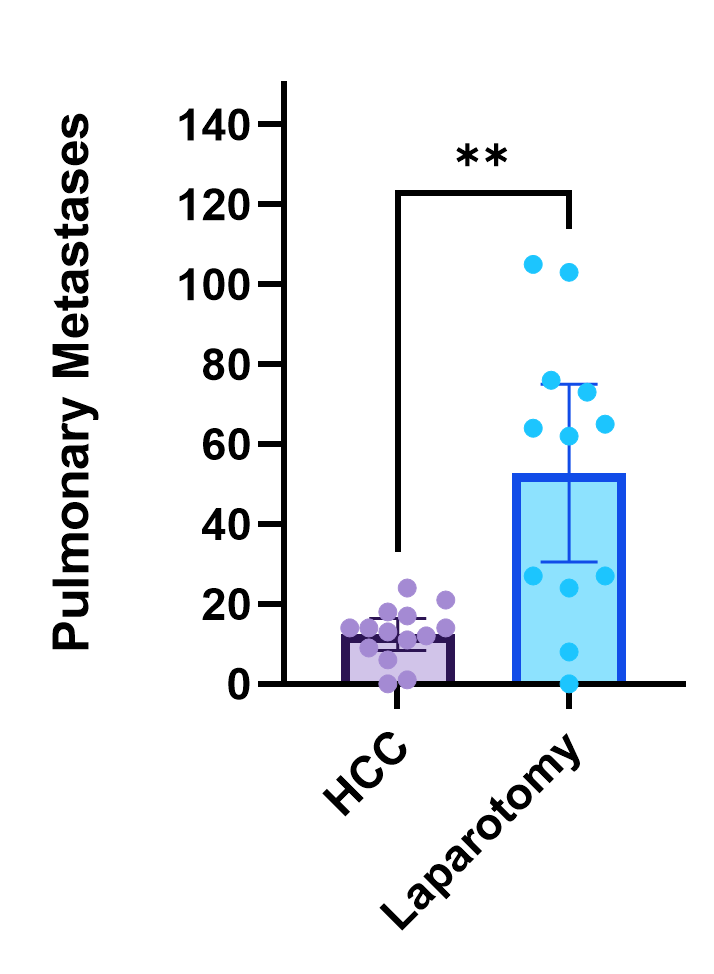


Laparotomy two hours before MADB106 tumor cell inoculation led to a significant increase (p = 0.002) in the number of pulmonary metastases (M = 52.833) compared to the non-operated control group (M = 12.429). Error bars represent 95% CI.
